# Supplementary figures and images for: Sex chromosomes control vertical transmission of feminizing Wolbachia symbionts in an isopod
Source: PLoS Biol. 2019 Oct 10;17(10):e3000438. doi: 10.1371/journal.pbio.3000438 (PMC6805007; doi:10.1371/journal.pbio.3000438)

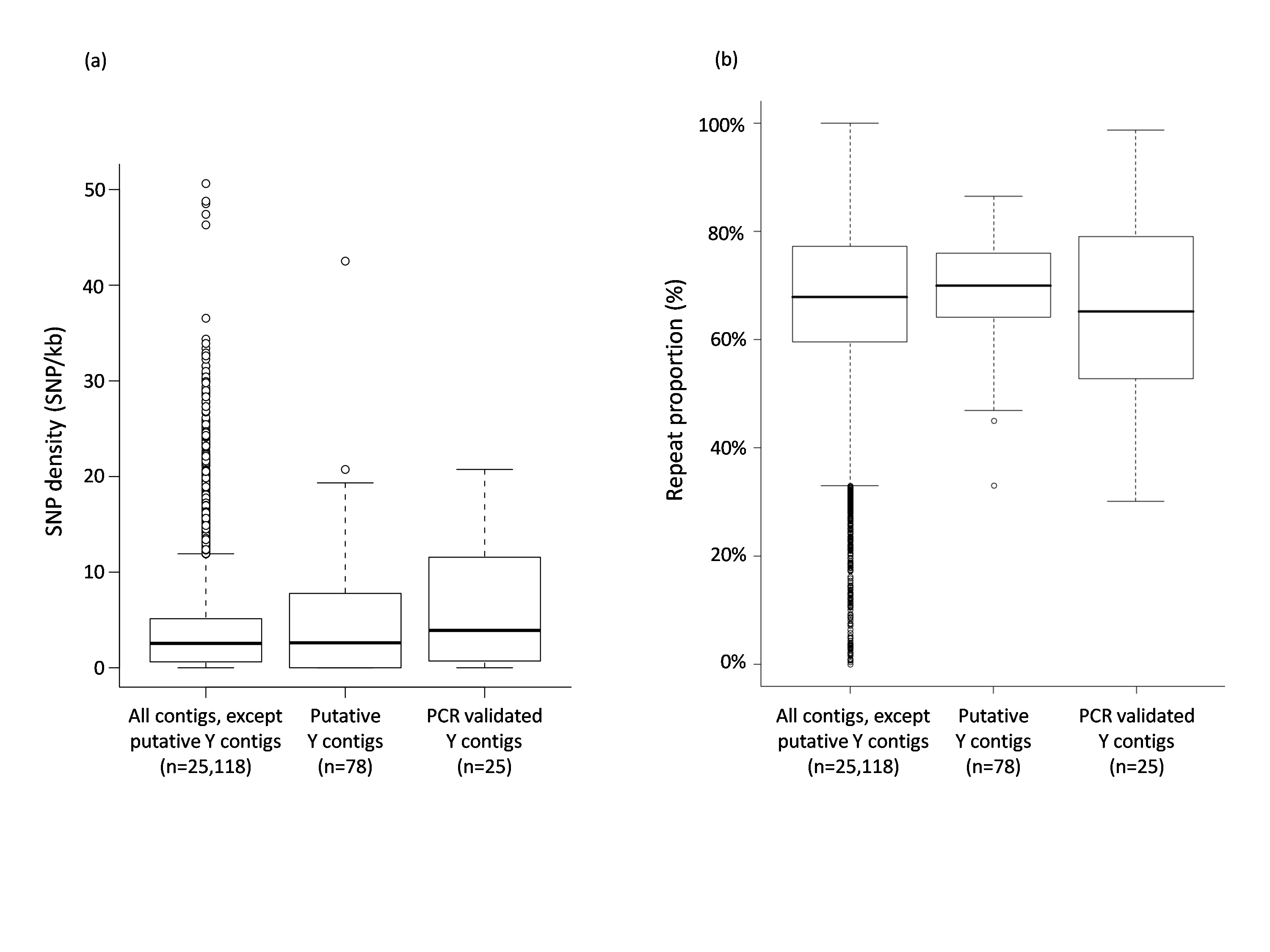

Supplement: S1 Fig — Box plots of SNP density (a) and repeat proportion (b) in the 78 computationally inferred, putative Y-linked contigs, a subset of 25 of the 78 contigs that were independently validated as Y-linked by PCR, and all the other contigs of the assembly. Thick lines and boxes depict median and interquartile range, respectively. Whiskers are bounded to the most extreme data point within the 1.5 interquartile range. Open circles represent outliers. The underlying data for this figure can be found in S6 Table. SNP, single nucleotide polymorphism. (TIF) [file pbio.3000438.s001.tif]

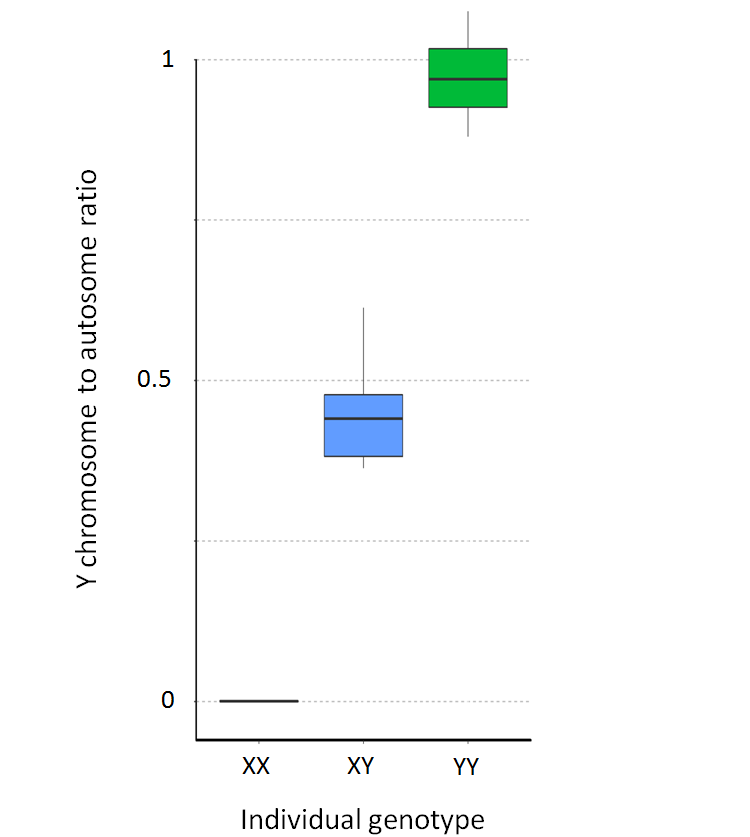

Supplement: S2 Fig — Y chromosome to autosome ratios were calculated for 60 individuals and compared with expected ratios: 1 for YY individuals (corresponding to 18 males and three females), 0.5 for XY individuals (28 males and five females), and 0 for XX individuals (six females). Thick lines and boxes depict median and interquartile range, respectively. Whiskers are bounded to the most extreme data point within the 1.5 interquartile range. (TIF) [file pbio.3000438.s002.tif]

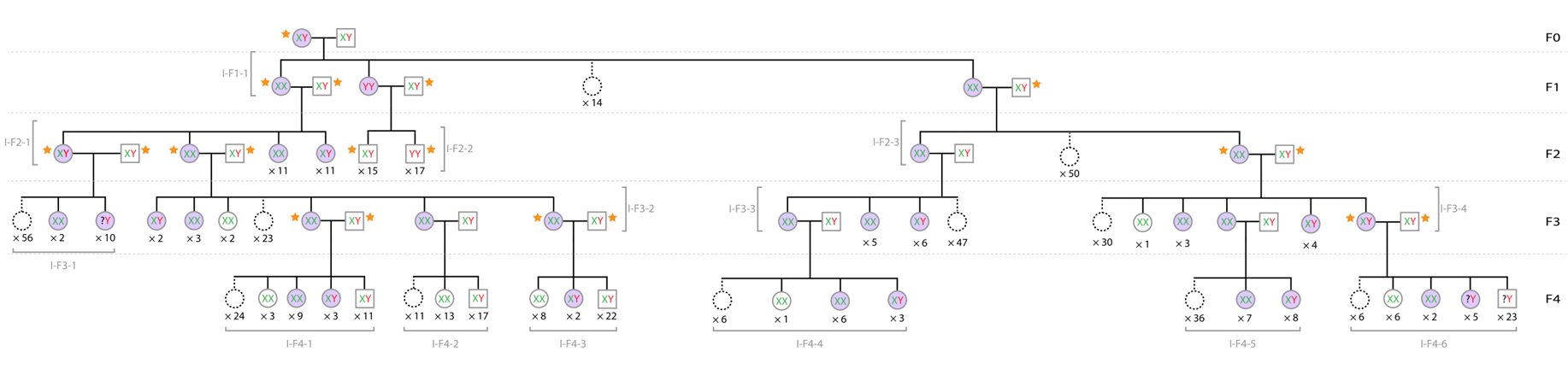

Supplement: S3 Fig — The pedigree spans five generations (F0–F4) and comprises 572 individuals (119 males and 453 females), 269 of which were included in molecular analyses (individuals not included in the molecular analyses are shown in dotted circles). Males are shown as squares, and females are shown as circles. Individuals carrying Wolbachia are shown in purple. Progeny identifiers are shown in gray. Sex chromosome genotype of individuals marked with an orange star was also assessed with a quantitative PCR assay. (TIF) [file pbio.3000438.s003.tif]

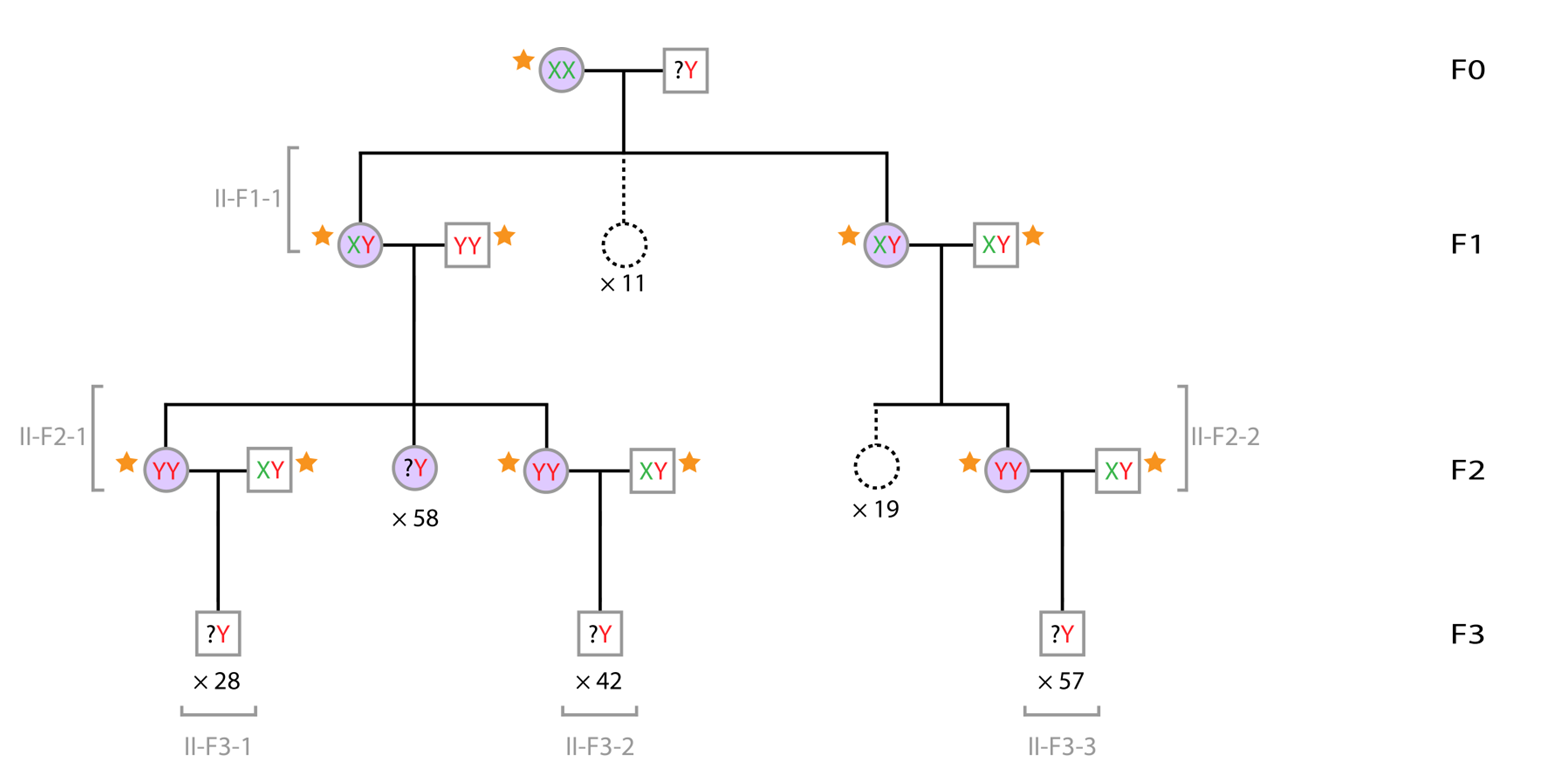

Supplement: S4 Fig — The pedigree spans four generations (F0–F3) and comprises 226 individuals (132 males and 94 females), 196 of which were included in molecular analyses (individuals not included in the molecular analyses are shown in dotted circles). Males are shown as squares, and females are shown as circles. Individuals carrying Wolbachia are shown in purple. Progeny identifiers are shown in gray. Sex chromosome genotype of individuals marked with an orange star was also assessed with a quantitative PCR assay. (TIF) [file pbio.3000438.s004.tif]

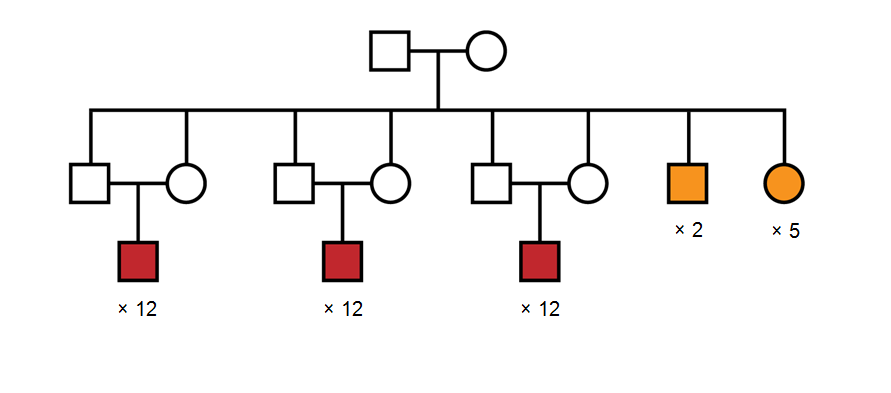

Supplement: S5 Fig — Males are shown as squares, and females are shown as circles. (TIF) [file pbio.3000438.s005.tif]

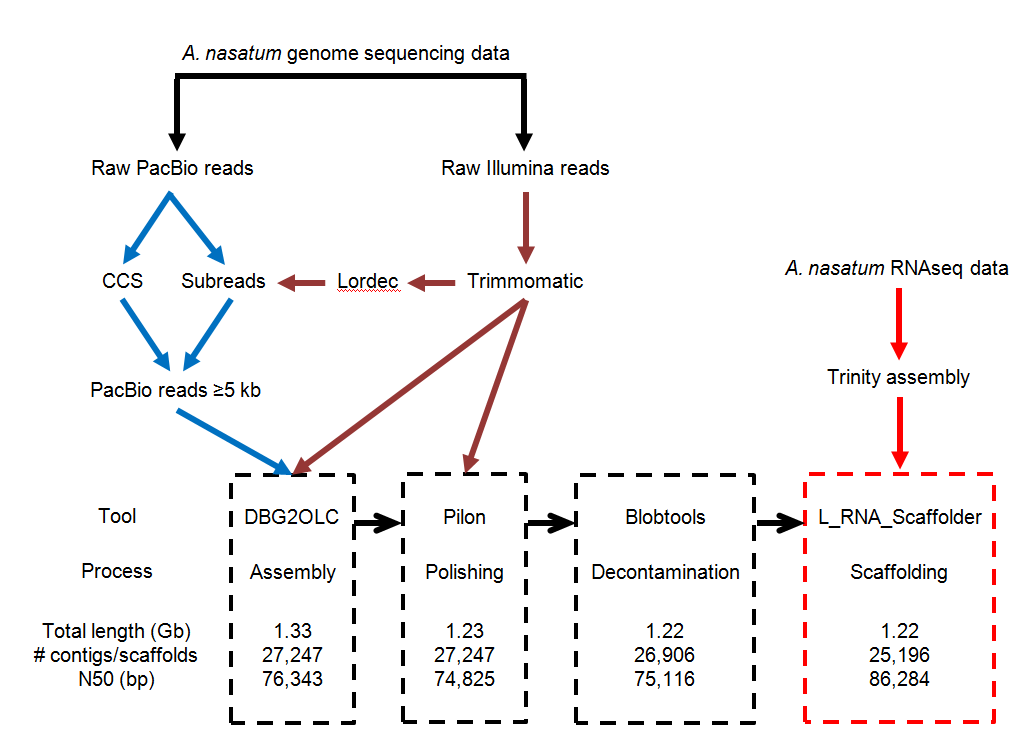

Supplement: S6 Fig — (TIF) [file pbio.3000438.s006.tif]
